# Dual-module aCAR-iCAR NK cells for solid tumors: cascade resistance mechanisms, AI-driven engineering, and precision stratification

**DOI:** 10.3389/fimmu.2026.1863734

**Published:** 2026-07-28

**Authors:** Chenru Ma, Yafei Zhuang, Songchen Han, Shuping Li, Qiang Dai

**Affiliations:** Department of Radiation Oncology, Gansu Provincial People’s Hospital, Lanzhou, Gansu, China

**Keywords:** aCAR-iCAR, artificial intelligence, CAR-NK cells, cascade resistance, dual-module CAR, NKG2A-HLA-E, precision immunotherapy, trogocytosis

## Abstract

Chimeric antigen receptor-engineered natural killer (CAR-NK) cells have emerged as a promising off-the-shelf platform for cancer immunotherapy, with a favorable safety profile in early clinical trials and potent antitumor activity demonstrated in relapsed/refractory (R/R) hematologic malignancies. However, their clinical efficacy in solid tumors remains severely limited by interconnected resistance mechanisms. In this review, we systematically dissect the pathological basis of CAR-NK therapy failure in solid tumors and propose an integrative cascade resistance framework that delineates three core bottlenecks: tumor microenvironment (TME)-mediated functional exhaustion, structural defects of non-natural killer (NK)-cell-adapted chimeric antigen receptors (CARs), and trogocytosis-driven immune escape. We further characterize the context-dependent regulatory roles of the natural killer group 2 member A–human leukocyte antigen E (NKG2A-HLA-E) immune checkpoint axis and the intercellular adhesion molecule 1/lymphocyte function-associated antigen 1 (ICAM-1/LFA-1) adhesion pathway within this cascade model, with clear stratification of evidence strength across all mechanistic conclusions. Centered on the activating-inhibitory dual-module CAR (aCAR-iCAR) system, we summarize its design principles, preclinical validation status, and potential to mitigate cascade resistance, with explicit distinction between killer cell immunoglobulin-like receptor (KIR)-based (Level 1 evidence) and NKG2A-based (Level 2–3 evidence) inhibitory CAR backbones. We then outline an end-to-end artificial intelligence (AI)-driven rational design framework covering target screening, structural optimization, and signaling balance calibration, and introduce the AI-nanosymbiont concept as an exogenous synergistic strategy to address TME delivery barriers. Building on the molecular heterogeneity of solid tumors, we propose a four-subtype precision stratification framework to match tumor features with tailored therapeutic regimens, and summarize core translational challenges including manufacturing constraints, regulatory gaps, and safety considerations. Overall, this review provides a balanced, evidence-graded theoretical framework for next-generation CAR-NK development against solid tumors, and generates testable hypotheses for future mechanistic and clinical investigations.

## 1 Introduction

In 2017, the U.S. Food and Drug Administration (FDA) approved the world’s first cluster of differentiation 19 (CD19)-targeted chimeric antigen receptor T-cell (CAR-T) therapy for clinical use, which has since revolutionized the treatment paradigm of relapsed/refractory (R/R) hematologic malignancies (1, 2). However, over the past decade, the clinical application of this therapy has been persistently limited by inherent drawbacks, including cytokine release syndrome (CRS), neurotoxicity, manufacturing constraints of autologous products, and its limited therapeutic efficacy against solid tumors (3–5).

Natural killer (NK) cells have emerged as a leading candidate platform for off-the-shelf immunotherapy, owing to their core advantages: major histocompatibility complex (MHC)-unrestricted target recognition, intrinsically low toxicity, and a negligible risk of graft-versus-host disease (GVHD) (6, 7). NK cells can be derived from a wide range of sources and exert dual anti-tumor effects via both CAR-dependent and innate cytotoxic pathways, endowing them with outstanding clinical translation value (8). Multiple clinical trials have validated the safety and efficacy of CAR-NK therapy. For instance, umbilical cord blood-derived CD19 CAR-NK cells achieved an objective response rate (ORR) of 73% in R/R B-cell malignancies with no grade ≥3 cytokine release syndrome, neurotoxicity, or graft-versus-host disease (GVHD) observed (9–11); and the NKG2D-targeted agent NKX101 has also delivered promising therapeutic outcomes in acute myeloid leukemia (AML) (12). Despite these advances, CAR-NK therapy exhibits a marked discrepancy in efficacy between hematologic and solid tumors, with cascade drug resistance, trogocytosis, and off-target toxicity remaining the core barriers to its clinical translation in solid tumors (13). The activating-inhibitory dual-module CAR (aCAR-iCAR) system, which remodels the signaling balance of NK cells, provides a promising technical strategy to overcome the aforementioned bottlenecks (14).

Few reviews have systematically stratified the evidence levels of AI-empowered dual-module CAR-NK strategies and integrated AI-nanosymbiont (AI-optimized nanodelivery) strategies to address solid tumor resistance cascades. To address this critical gap, this review systematically dissects the cascade pathological mechanisms underlying CAR-NK therapeutic failure in solid tumors and elucidates the molecular basis of the three core drug resistance bottlenecks, as well as the key nodes of immune escape. Furthermore, we provide an in-depth elaboration of the design rationale of the aCAR-iCAR system, the AI-empowered full-chain optimization framework, and the synergistic strategy based on nanodelivery systems. Finally, we propose a four-subtype precision treatment scheme. This work aims to provide systematic theoretical reference and practical insights for the clinical translation of CAR-NK immunotherapy for solid tumors.

This narrative review was conducted based on literature retrieved from PubMed, Web of Science, and ClinicalTrials.gov, with a search date range from January 2018 to December 2025. Key search terms included: CAR-NK, chimeric antigen receptor natural killer, aCAR-iCAR, dual-module CAR, AI-guided CAR engineering, nanomedicine CAR-NK delivery, and solid tumor CAR-NK resistance. We included original research articles, published clinical trial reports, and systematic reviews, all with complete experimental or clinical data; conference abstracts, non-English literature, review protocols without substantive data, and retracted publications were excluded. Clinical trials were identified independently via ClinicalTrials.gov (as of December 31, 2025), and only interventional studies with full registered records were included for landscape analysis.

## 2 Cascading pathological mechanisms of CAR-NK therapy failure in solid tumors

As outlined in the Introduction, CAR-NK therapy has demonstrated clear efficacy and a favorable safety profile in relapsed/refractory (R/R) hematologic malignancies, yet its clinical translation for solid tumors faces formidable challenges. To systematically characterize the current research landscape, as detailed in the Introduction, we identified 122 eligible CAR-NK clinical trials from ClinicalTrials.gov (as of December 31, 2025), of which 41 (33.6%) focused on solid tumors — a proportion markedly lower than that for hematologic malignancies. This proportion is consistent with data from a previous systematic review of the global CAR-NK clinical landscape (15).

Accumulating evidence shows that this translational gap is associated with three interconnected pathological processes and multiple immune escape pathways. Building on independent preclinical and clinical findings, we propose an integrative cascade resistance framework with potential vicious cycle amplification effects, where three key nodes act at different stages of therapeutic failure: tumor microenvironment (TME)-mediated functional exhaustion as a proposed initiating driver, structural defects of non-NK-cell-adapted CAR as a central amplifying hub, and trogocytosis-mediated immune escape as a key terminal bottleneck (16, 17).

While the individual contributions of these nodes are established, their potential to form a self-reinforcing cascade highlights the need for multi-faceted therapeutic interventions, as will be discussed in later sections.

### 2.1 TME-mediated multiple barriers and NK cell dysfunction: the initiating factor of the cascading reaction

The physical and immune barriers established by the solid tumor TME represent the primary obstacle to the effector function of CAR-NK cells, as well as the initiating factor of the cascading reaction.

Resistance of solid tumors to CAR-NK cell therapy is triggered by the dual blockade of physical and immune barriers (18). At the physical level, the dense extracellular matrix (ECM), aberrant tumor vasculature, and elevated interstitial fluid pressure collectively impede the infiltration of intravenously infused CAR-NK cells into the tumor parenchyma (19, 20). Consequently, only a very small fraction of effector cells can reach the tumor site, failing to achieve an effective tumoricidal concentration.

At the immune level, regulatory T cells (Tregs), M2-polarized tumor-associated macrophages (TAMs), and myeloid-derived suppressor cells (MDSCs) enriched in the TME form a coordinated suppressive network. These cells impair CAR-NK cell function via three primary mechanisms: (1) secretion of immunosuppressive cytokines such as transforming growth factor-β (TGF-β) and interleukin-10 (IL-10); (2) competitive depletion of essential nutrients; and (3) release of immunosuppressive metabolites including adenosine, lactic acid, and reactive oxygen species (ROS) (21–23). These effects collectively disrupt the metabolic homeostasis of NK cells and block activating signal transduction, thereby driving CAR-NK cell dysfunction. This dysfunctional state, characterized by global impairments in proliferation, cytokine secretion, and tumoricidal capacity, represents the core mechanism underlying the rapid *in vivo* loss of function of CAR-NK cells that exhibit robust effector activity *in vitro* (24, 25).

Under chronic suppressive pressure within the TME, the expression of inhibitory receptors on the surface of NK cells, such as killer cell lectin-like receptor subfamily C member 1 (KLRC1/NKG2A), is upregulated (26, 27). Meanwhile, tumor cells upregulate the expression of human leukocyte antigen E (HLA-E) ligands, thereby potentiating the inhibitory signaling of the NKG2A-HLA-E axis (28, 29). This positive feedback loop can drive a vicious cycle of amplified inhibitory signals and exacerbated dysfunction; however, the regulatory strength of this axis is not universal — it varies substantially across tumor types, HLA-E expression levels, peptide loading profiles, and immune microenvironment contexts. In addition, chemokine ligand-receptor mismatch in the TME reduces the infiltration efficiency of CAR-NK cells (30). Suppressive signals such as TGF-β in the TME can induce epigenetic reprogramming in NK cells (31). The specific mechanisms underlying these epigenetic alterations may include aberrant modifications mediated by DNA methyltransferases (e.g., DNMT3A), and such events are widely thought to collectively drive NK cell dysfunction.

Additionally, downregulation of intercellular adhesion molecule 1 (ICAM-1) on tumor cells, a well-documented immune evasion mechanism that impairs lymphocyte function-associated antigen 1 (LFA-1)-dependent immunological synapse formation in endogenous NK cells, may be further amplified within the CAR-NK cascade resistance framework, as will be analyzed in Section 2.4.

Taken together, the multiple TME-mediated suppressive effects not only directly impair the antitumor effector function of CAR-NK cells, but also lower their response threshold to CAR activation signals, creating permissive conditions for the subsequent development of CAR structural defects and trogocytosis. Thus, these diverse TME-mediated suppressive effects serve as the initiating link of this cascading pathological process.

### 2.2 Structural defects of non-NK cell-adapted CAR: the central hub of the cascading vicious cycle

The CAR structures of clinical-stage CAR-NK products are largely directly carried over from CAR-T platforms, without adaptive optimization for the intrinsic lineage-specific differences in the signal transduction networks between NK cells and T cells. This is a core intrinsic driver of the functional defects of CAR-NK cells in solid tumors (32). The classical CAR-T structure adopts a signaling module combining a CD28 or 4-1BB co-stimulatory domain with CD3ζ (CD3 zeta, CD3Z). This design fails to fully leverage NK cell-specific endogenous signaling axes such as DAP10/DAP12 and 2B4, and thus may not optimally activate the effector programs unique to NK cells (33, 34). This adaptation defect triggers a series of chain reactions, manifested as insufficient CAR-mediated NK cell activation, reduced *in vivo* persistence, and elevated risk of off-target toxicity. These effects further amplify TME-mediated cellular dysfunction and the likelihood of trogocytosis, establishing this structural defect as the central hub linking the extrinsic immune barriers of tumors to terminal therapeutic failure (35). In addition, design defects in the single-chain variable fragment (scFv) of conventional CARs readily induce tonic baseline signaling, which exacerbates NK cell dysfunction while increasing the risk of non-specific activation and trogocytosis, ultimately forming a secondary pathological amplification effect mediated by structural defects (36, 37).

### 2.3 Trogocytosis-mediated antigen loss and effector cell self-destruction: a terminal bottleneck of variable clinical dominance

Trogocytosis is a well-characterized biological phenomenon wherein tumor target antigens are transferred to the effector cell membrane during immunological synapse formation between CAR-NK cells and tumor cells. While this effect has a limited functional impact in hematologic malignancies, it can be markedly amplified by matrix stiffening and the hypoxic microenvironment within the solid tumor TME, rendering it a key terminal bottleneck mediating CAR-NK therapy failure in solid tumors.

Notably, the contribution of trogocytosis to therapeutic resistance is not uniformly dominant across all clinical scenarios, but is regulated by multiple molecular, cellular and microenvironmental factors.

#### 2.3.1 Molecular determinants and cellular mechanisms of trogocytosis

The magnitude of trogocytosis is jointly regulated by multiple molecular factors at the immunological synapse. Antigen density on tumor cells is positively correlated with membrane transfer efficiency: higher expression of target antigens provides more substrates for CAR-mediated synapse formation and internalization, significantly increasing the frequency and intensity of trogocytic events (32, 38). CAR affinity is a critical modulator of this process: high-affinity scFv domains paradoxically accelerate antigen stripping from tumor cell membranes and increase the probability of fratricide between effector cells, while rational affinity tuning can reduce trogocytosis risk without proportional loss of cytotoxic potency (37). Furthermore, synapse duration—governed by CAR signaling strength and cytoskeletal remodeling—determines the time window for membrane fragment transfer. Prolonged synaptic contact directly increases the amount of antigen acquired by effector cells, a phenomenon well-documented in both endogenous and CAR-engineered NK cells (32, 39).

At the cellular level, trogocytosis blocks the antitumor immune response primarily via two pathways. On the tumor cell side, trogocytosis drives sustained target antigen loss, inducing antigen-dependent immune escape and reducing the recognition efficiency of subsequent effector cells (13, 17). On the effector cell side, acquired tumor antigens on the NK cell surface trigger distinct detrimental outcomes. One proposed mechanism is antigen masking, where transferred antigens may sterically hinder the CAR’s antigen-binding site in cis, attenuating further activation. More critically, and supported by direct experimental evidence, fratricide occurs when neighboring CAR-NK cells recognize the acquired tumor antigens on adjacent effector cells and initiate cytotoxic killing, resulting in a sharp decline in functional effector cell numbers and progressive abrogation of overall tumoricidal activity (14, 39).

#### 2.3.2 TME-dependent amplification of trogocytosis

In solid tumors, the unique physicochemical properties of the TME markedly amplify trogocytosis and its downstream detrimental effects. Matrix stiffening, a hallmark of desmoplastic solid tumors, increases mechanical tension at the immunological synapse, which can facilitate membrane exchange and accelerate antigen transfer efficiency (19, 20). The hypoxic and acidic metabolic microenvironment further exacerbates NK cell metabolic stress, lowers the activation threshold for activation-induced cell death after trogocytosis, and sensitizes effector cells to fratricidal killing (24, 25).

These microenvironmental factors form a self-reinforcing vicious cycle: trogocytosis-mediated effector cell depletion reduces the overall effector-to-target (E:T) ratio at the tumor site; a lower E:T ratio forces surviving CAR-NK cells to form synapses more frequently with individual tumor cells, paradoxically increasing per-cell trogocytic events and accelerating functional exhaustion (13, 17). This E:T ratio dependency is a key distinction between solid tumors and hematologic malignancies: in hematologic malignancies, effector cells can rapidly access circulating tumor cells and are continuously replenished, while in solid tumors, poor infiltration and physical stromal trapping inherently limit the achievable E:T ratio, greatly amplifying the functional impact of trogocytosis-mediated cell loss (18, 35).

#### 2.3.3 Clinical relevance and contextual dominance of trogocytosis

The clinical dominance of trogocytosis as a resistance mechanism is highly context-dependent. It is most likely to be the primary driver of therapeutic failure when three conditions coexist: high tumor antigen density, high CAR affinity, and limited CAR-NK infiltration resulting in a low intratumoral E:T ratio (37).

Conversely, in scenarios where CAR affinity is rationally optimized, tumor antigen density is moderate and heterogeneous, or where high intratumoral E:T ratios can be achieved and maintained (e.g., via local intratumoral infusion), trogocytosis plays a less dominant role. In these contexts, TME-mediated functional exhaustion or CAR structural signaling defects become the primary bottlenecks limiting therapeutic efficacy (32). This variable dominance is the core rationale for defining trogocytosis as a “terminal bottleneck”: it does not universally dominate resistance, but when it occurs on the basis of pre-existing TME dysfunction and suboptimal CAR signaling, it can trigger irreversible collapse of the antitumor immune response (17, 35).

#### 2.3.4 Mechanistic rationale for iCAR-mediated blockade of fratricide

Based on the above mechanisms, the inhibitory CAR (iCAR) design is proposed to interrupt the fratricide pathway by leveraging NK cell self-recognition machinery. NK cells natively express high levels of self-antigens (including human leukocyte antigen class I (HLA-I) molecules and HLA-E) on their surface (14, 26). When CAR-NK cells acquire tumor antigens via trogocytosis, these endogenous self-antigens remain stably expressed on the effector cell membrane.

An iCAR targeting self-antigens can be engaged in cis (on the same cell) or in trans (on neighboring trogocytotic cells) to deliver immunoreceptor tyrosine-based inhibitory motif (ITIM)-mediated inhibitory signals, which override the activating signals triggered by acquired tumor antigens and block the cytotoxic program of fratricide (14). For KIR-based iCARs targeting classical HLA class I molecules, this fratricide-blocking effect has been directly validated with quantitative experimental data, confirming that dual-module CAR-NK cells maintain high survival rates and intact tumoricidal activity after long-term co-culture with tumor cells. For NKG2A-based iCARs targeting HLA-E, this protective mechanism remains mechanistically plausible based on endogenous NK cell checkpoint biology, but has not yet been systematically confirmed in CAR-NK engineering systems (26).

By reducing effector cell loss, iCARs can partially disrupt the trogocytosis-driven vicious cycle. This helps alleviate NK cell dysfunction and attenuate TME-mediated immunosuppression, thereby interrupting cascade resistance at its terminal node. This represents a key mechanistic basis for dual-module CAR-NK cells to overcome solid tumor resistance.

### 2.4 Cascade amplification effect of the three core bottlenecks and full-dimensional therapeutic barriers

Based on accumulated preclinical and clinical evidence, we propose an integrative cascade resistance framework to interpret the overall pathological process of CAR-NK therapeutic failure in solid tumors. To clarify the strength of supporting evidence, we stratify the relevant conclusions into three levels:

#### 2.4.1 Level 1: independently validated pathological mechanisms (experimental evidence)

The three core bottlenecks described above have all been independently confirmed by multiple *in vitro* and *in vivo* studies:

1. TME-mediated NK cell dysfunction, including metabolic repression, inhibitory receptor upregulation and functional exhaustion, has been widely verified in various solid tumor models and clinical samples (18, 24, 26).

2. Signaling defects caused by directly transplanted CAR-T-derived CAR structures in NK cells, including insufficient activation signal and poor *in vivo* persistence, have been validated by comparative studies of different CAR intracellular domains (32, 33).

3. Trogocytosis-mediated antigen loss and effector cell fratricide have been directly observed in co-culture systems and *in vivo* tumor models, and are recognized as a key mechanism of CAR-NK failure in solid tumors (14, 17).

#### 2.4.2 Level 2: plausible cross-node interactions (indirect and correlative evidence)

The interactive and amplifying relationships between the three core bottlenecks are reasonable mechanistic deductions based on existing research conclusions, but lack direct serial verification experiments covering multiple nodes.

For example, TME-induced metabolic reprogramming can reduce the activation threshold of CAR signaling and exacerbate the functional defects of non-optimized CAR structures (23, 36); aberrant tonic signaling caused by CAR structural defects can in turn accelerate NK cell exhaustion and increase the probability of trogocytosis (35, 37).

Therefore, while these links are mechanistically plausible and form a strong basis for our model, they should be viewed as hypotheses that require dedicated validation.

#### 2.4.3 Level 3: the integrative cascade resistance model (proposed framework)

Integrating the confirmed independent mechanisms (Level 1) and the plausible crosstalk (Level 2), we formulate a testable, closed-loop cascade amplification model in which TME-mediated cellular dysfunction serves as the initiating factor, structural defects of non-NK cell-adapted CAR act as the hub for pathological amplification, and trogocytosis-mediated antigen loss and effector cell depletion act as the terminal bottleneck of therapeutic failure—with its clinical dominance varying across tumor contexts as detailed in Section 2.3. These three factors form a potential vicious cycle: immunosuppression exacerbates CAR functional defects, structural defects amplify cellular dysfunction, and therapeutic failure further promotes the deterioration of the immunosuppressive TME.

In addition, impaired immunological synapse formation caused by ICAM-1 downregulation on tumor cells may further accelerate this pathological process (40). While ICAM-1/LFA-1-dependent synapse biology is a well-established mechanism governing endogenous NK cell cytotoxicity, its integration into the cascade resistance framework of CAR-NK cells, and its functional interaction with aCAR signaling to bypass adhesion-dependent activation, represent a novel conceptual synthesis for understanding solid tumor resistance to CAR-NK therapy (41).

Dysregulated activation of the NKG2A-HLA-E axis may also amplify cascade resistance in tumors with high HLA-E expression, but its contribution is context-dependent rather than universal.

Together with the three core bottlenecks, these mechanisms constitute multi-dimensional therapeutic barriers, which may explain why localized intervention strategies such as single-target CAR optimization and monotherapy with immune checkpoint inhibitors—including both T-cell and NK-cell checkpoint blockade—have failed to achieve substantive therapeutic breakthroughs in solid tumors.

Overall, this proposed cascade resistance framework suggests that it is difficult to completely reverse therapeutic failure only through localized optimization of a single target. Therefore, there is an urgent unmet need to develop integrated cell engineering strategies that can simultaneously overcome multiple pathological barriers.

**Figure 1:**
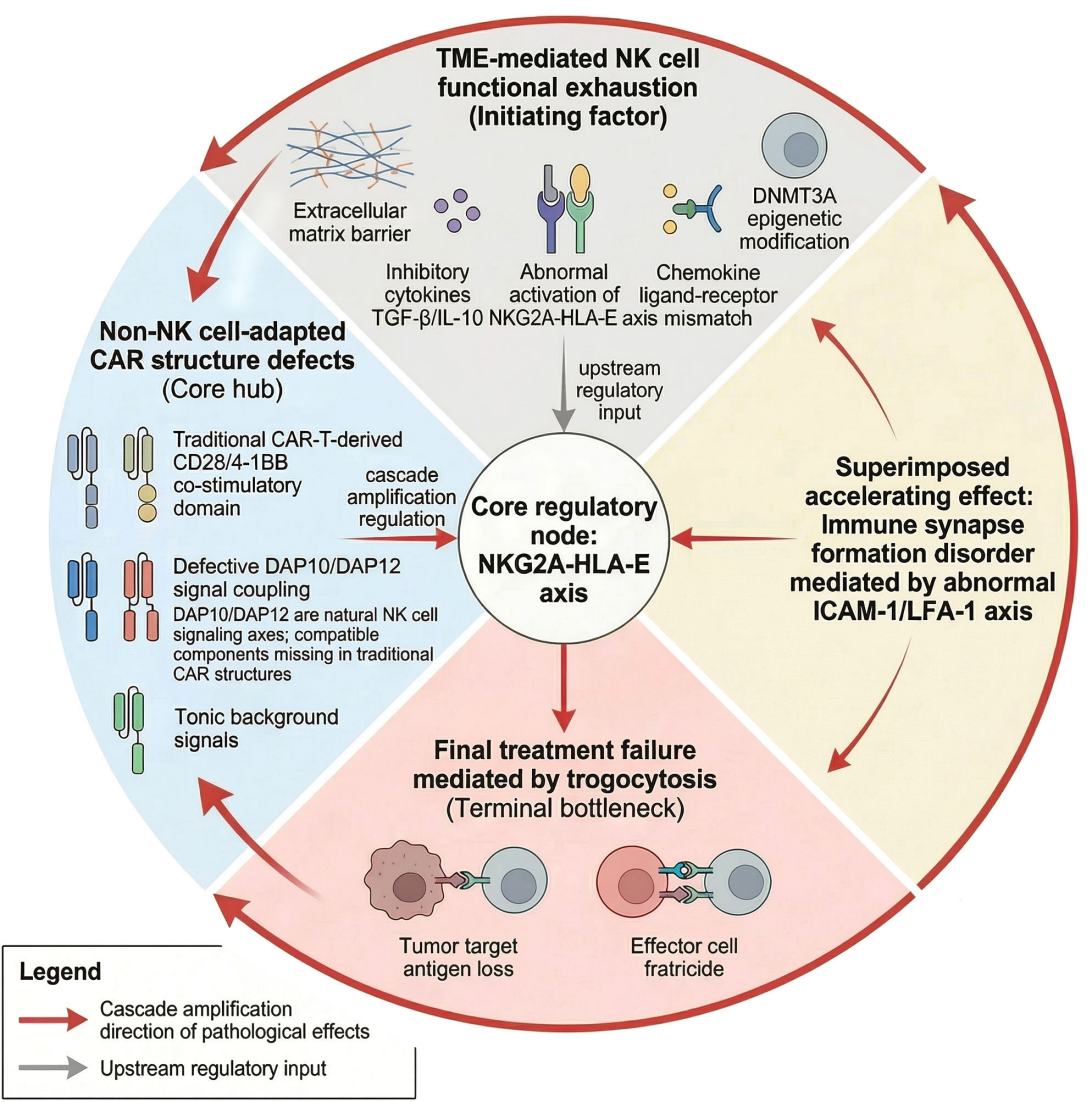
The closed-loop vicious cycle of cascade resistance in CAR-NK therapy for solid tumors. This image systematically illustrates the three core bottlenecks driving CAR-NK therapy failure in solid tumors and their vicious cycle of cascade amplification: ① TME-mediated NK cell functional exhaustion and infiltration barrier, including extracellular matrix barrier, inhibitory cytokine (TGF-β/IL-10) enrichment, abnormal activation of the NKG2A-HLA-E axis, chemokine ligand–receptor mismatch, and DNMT3A-mediated epigenetic modification, function as the initiating driver of the cascade reaction; ② Non-NK cell-adapted CAR structure defects, which cannot couple with the endogenous DAP10/DAP12 signal axis of NK cells and induce tonic background signals, are the core hub that amplifies all pathological effects; ③ Trogocytosis-mediated tumor target antigen loss and effector cell fratricide are the terminal bottleneck leading to final treatment failure. The NKG2A-HLA-E axis is the core regulatory node of the whole cascade reaction, and the immune synapse formation disorder mediated by abnormal ICAM-1/LFA-1 axis further superimposes and accelerates the progression of the cascade vicious circle. Legend: Red arrows indicate the direction of pathological cascade amplification; gray arrows indicate upstream regulatory input to core node. CAR-NK, chimeric antigen receptor-engineered natural killer; TME, tumor microenvironment; TGF-β, transforming growth factor-β; IL-10, interleukin-10; DNMT3A, DNA methyltransferase 3 alpha; ICAM-1, intercellular adhesion molecule 1; LFA-1, lymphocyte function-associated antigen 1. This image illustrates the proposed cascade resistance framework with potential sequential amplification effects.

The cascade resistance framework and corresponding evidence levels are summarized in **Figure 1**.

## 3 Activation-inhibition dual-module CAR system: the core technical system to overcome cascade therapeutic bottlenecks of solid tumors

Based on the aforementioned pathological characteristics of cascaded drug resistance, the aCAR-iCAR dual-module system provides a proposed core technical solution for breaking through the therapeutic bottlenecks of solid tumors via precise regulation of signaling balance. Localized intervention at a single link cannot break the vicious cycle of cascaded drug resistance, so there is an urgent unmet need to establish a systematic strategy that can simultaneously overcome the three core bottlenecks and immune escape. The aCAR-iCAR dual-module system is currently the most promising design with the greatest potential for clinical translation, but the level of experimental validation varies substantially across different iCAR backbones. This chapter systematically elaborates the design principles, functional breakthroughs, and application boundaries of this system, laying a theoretical foundation for subsequent artificial intelligence-empowered rational design.

### 3.1 Design principles and functional breakthroughs of the aCAR-iCAR dual-module CAR system

The aCAR-iCAR system is constructed based on the innate “missing self” recognition mechanism of NK cells. It represents a proposed technological paradigm shift of CAR-NK from a “single activation switch” to a modular signaling regulator, and is expected to simultaneously address the three core cascaded drug resistance bottlenecks in solid tumors (14).

#### 3.1.1 Core design principles

The activation status of NK cells is governed by the net signaling balance between activating and inhibitory receptors, which constitutes the most fundamental biological distinction between NK cells and T cells. The dual-module CAR system recapitulates this innate regulatory mechanism and consists of two core functional modules:

Activating CAR (aCAR): It adopts the canonical structure composed of an scFv, a hinge region, a transmembrane domain, and an intracellular signaling domain, and specifically targets tumor-associated antigens (TAAs). Its intracellular domain integrates the NK cell-specific 2B4/DAP12-CD3ζ signaling axis, which delivers robust activating signals exclusively upon tumor antigen recognition, thereby partially mitigating the non-adaptive structural defect of conventional CAR-derived designs (33, 42). This potent activating signal exerts dual modulatory effects on CAR-NK cell function: it bypasses the co-stimulatory requirement of LFA-1, and antagonizes inhibitory signaling downstream of the NKG2A-HLA-E immune checkpoint axis. These effects collectively enable CAR-NK cells to form stable immunological synapses and exert robust cytotoxicity against target cells even in the absence of ICAM-1/LFA-1 engagement, thereby counteracting a well-characterized mechanism of tumor immune escape to a certain extent (40).

Inhibitory CAR (iCAR): iCARs are composed of an scFv targeting self-antigens on normal tissues, fused to the transmembrane domain of an NK inhibitory receptor and an ITIM.

Two main design backbones have been proposed, with distinct levels of experimental validation:

- KIR-based iCARs (Level 1 evidence): This design targets classical HLA-I molecules and is supported by direct *in vivo* experimental evidence from the 2022 *Nature Medicine* study. It follows the classic “missing self” principle: when tumor cells lose HLA-I expression (HLA-I loss of heterozygosity, LOH), iCAR is not engaged, allowing aCAR-mediated killing; in normal tissues with intact HLA-I, iCAR delivers inhibitory signals to suppress off-target toxicity (14).

- NKG2A-based iCARs (Level 2–3 evidence): This design targets HLA-E molecules and is largely conceptually extrapolated from NKG2A-HLA-E checkpoint biology, with very limited direct functional validation in CAR-NK systems. NKG2A forms a functional heterodimer with CD94 to recognize HLA-E peptides; its ITIM motifs enable tunable inhibitory signal transduction. Notably, monoclonal antibodies targeting NKG2A have completed clinical safety evaluation, whereas the NKG2A-iCAR platform itself remains at the early preclinical proof-of-concept stage (26, 43).

This design strictly adheres to the regulatory principles of the AND-NOT Boolean logic gate, triggering the cytotoxic program only under the condition of “target antigen-positive and self-antigen-negative”, and is proposed to simultaneously address multiple dimensions of therapeutic barriers in solid tumors (44).

#### 3.1.2 Four core functional breakthroughs

##### 3.1.2.1 Reduced off-target toxicity

Self-antigens on the surface of normal tissues can activate the inhibitory signals of iCAR, attenuate aberrant killing of normal cells by CAR-NK cells, and are proposed to widen the therapeutic window. Among different iCAR backbones, NKG2A-based iCARs (Level 2–3 evidence) enable precise recognition of HLA-E and theoretically release inhibition only when HLA-E expression is downregulated on tumor cells, which is expected to further improve the targeting specificity of the therapy (45, 46). Of note, KIR-based iCARs (Level 1 evidence) have been experimentally validated to reduce off-target toxicity *in vivo*.

##### 3.1.2.2 Blockade of trogocytosis and antigen escape

NK cells natively express high levels of HLA-I/HLA-E. When CAR-NK cells acquire tumor target antigens via trogocytosis, iCAR can deliver inhibitory signals to substantially inhibit effector cell fratricide, consistent with the trogocytosis-mediated resistance mechanism detailed in Section 2.3. For KIR-based dual-module CAR-NK systems (Level 1 evidence), *in vitro* experiments have directly validated this effect: after 72 h of co-culture with tumor cells, the survival rate of effector cells increased from 28% to 92%, with largely preserved tumoricidal activity (14, 47, 48).

##### 3.1.2.3 Reversal of TME-mediated dysfunction

Consistent with the TME-mediated NK cell dysfunction mechanism described in Section 2.1, the NK cell-specific signaling domain of aCAR may enhance the activation capacity and dysfunction resistance of CAR-NK cells, which exerts a potential synergistic effect with immune checkpoint inhibitors (ICIs). Meanwhile, it may upregulate the expression of chemokine receptors and partially improve insufficient tumor infiltration of CAR-NK cells (49). This effect can be further enhanced via cis-acting functional element engineering, as detailed in Section 3.2.2.

##### 3.1.2.4 Overcoming ICAM-1 downregulation-mediated synaptic impairment

Upon antigen recognition, aCAR (Level 1 evidence) supports the formation of stable ICAM-1-independent immunological synapses, which partially compensate for the LFA-1-mediated signaling pathway. This effect addresses the immune synapse defect described in the cascade resistance framework in Section 2.4. In ICAM-1 knockout preclinical tumor models, the killing efficiency of dual-module CAR-NK cells was reported to remain above 90%, which exhibited a notable advantage over conventional antibody-dependent cellular cytotoxicity (ADCC) therapy (50).

It is worth noting that addressing the dense stromal barrier of solid tumors may benefit from combination with AI-optimized nanodelivery systems (referred to as AI-nanosymbionts in this review), which are proposed to exert potential synergistic anti-tumor effects, as detailed in Section 5.2.

#### 3.1.3 AI-optimized three-tier safety switch system

Off-target toxicity remains one of the most critical safety liabilities in the clinical translation of CAR-based therapies. In this review, we propose an AI-optimized three-tier safety switch framework to achieve a potential balance between antitumor efficacy and therapeutic safety.

##### 3.1.3.1 Physiological safety switch

This switch is centered on the iCAR module (Level 1 evidence for KIR-based iCARs), and mediates physiological blockade of off-target toxicity via specific recognition of self-antigens on normal cells (14).

##### 3.1.3.2 Drug-responsive safety switch

AI-optimized pH-responsive nanodelivery systems (Level 2 evidence) can be applied to deliver CAR inhibitors. The agents are preferentially released in the blood circulation at physiological pH, which may support rapid management of acute toxicity without compromising therapeutic efficacy within the TME (24). The design principles of AI-optimized nanocarriers are further elaborated in Section 5.

##### 3.1.3.3 Depletable suicide switch

The system integrates an inducible suicide element (Level 2 evidence, validated in CAR-T clinical settings), which allows for rapid depletion of CAR-NK cells *in vivo* via exogenous drug administration, serving as a potential final safeguard for therapeutic safety (43).

AI technology is proposed to support synergistic optimization of these three classes of safety switches to construct a multi-layered toxicity control system, which may strengthen the safety basis for the clinical translation of this therapeutic strategy. Detailed discussion of clinical safety and regulatory requirements is provided in Section 6.2.

#### 3.1.4 Preclinical validation and existing challenges

In 2022, Li et al. completed the first systematic *in vivo* validation of the KIR-based dual-module CAR-NK system (Level 1 evidence) in *Nature Medicine*, and demonstrated that this design significantly enhanced the *in vivo* persistence of cells, prolonged the survival of tumor-bearing mice, and effectively mitigated off-target toxicity and trogocytosis-mediated fratricide (14, 48). Consistent with the *in vitro* findings detailed in Section 3.1.2.2; subsequent studies from 2023 to 2025 further revealed that this KIR-based design exhibited stable and robust antitumor activity in both solid tumor models with HLA class I loss of heterozygosity (LOH) and acute myeloid leukemia (AML) models (46). In contrast, NKG2A-based dual-module CAR-NK systems remain at the early preclinical proof-of-concept stage (Level 2–3 evidence), with no systematic *in vivo* efficacy validation reported to date.

For KIR-based dual-module systems with well-validated preclinical efficacy, the broader dual-module design paradigm still faces three core challenges in clinical translation. First, the heterogeneity of tumor HLA class I/HLA-E expression is prone to inducing immune escape, among which HLA-E heterogeneity represents a major bottleneck restricting the broad application of NKG2A-based iCARs, supporting the need for AI-based target screening to achieve precise patient stratification (43). Relevant AI screening strategies are further elaborated in Chapter 4. Second, given that the functional cell surface expression of NKG2A (51), depends on the formation of a heterodimer with the CD94 molecule via disulfide bonds, exogenous NKG2A-based iCARs, which requires heterodimerization with endogenous CD94 for surface expression, may compete with native NKG2A for CD94 binding. Furthermore, overexpression of these complexes could potentially lead to tonic inhibitory signaling independent of ligand engagement, which may dampen basal NK cell activity. Third, the system involves millions of parameter combinations, and the traditional univariate trial-and-error approach has extremely low optimization efficiency, highlighting the potential of AI-driven high-throughput rational design to achieve global optimization (24, 52).

**Figure 2:**
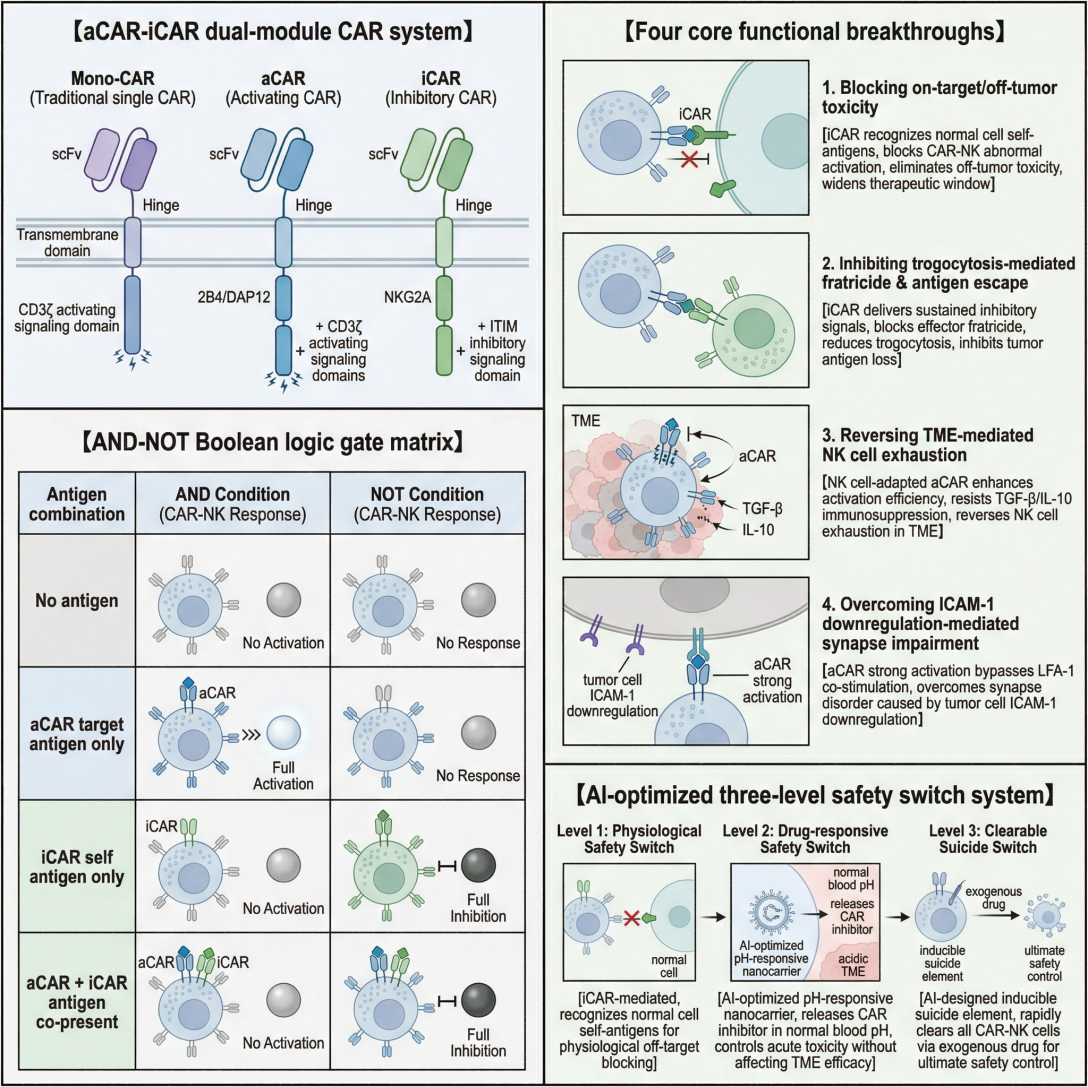
Structural design, logical regulation, functional breakthroughs and safety control system of the aCAR-iCAR dual-module CAR. This image systematically demonstrates the core design principle and application advantages of the aCAR-iCAR system, the key technology to overcome the cascade drug resistance of CAR-NK therapy in solid tumors. The top-left panel shows the structural comparison between traditional single CAR (Mono-CAR) and aCAR-iCAR dual-module CAR, in which aCAR is coupled with NK cell-specific 2B4/DAP12-CD3ζ activating signaling domains, and iCAR is fused with NKG2A-ITIM inhibitory signaling domains. The bottom-left panel shows the AND-NOT Boolean logic gate matrix of the aCAR-iCAR system, which clarifies the activation/inhibition response of CAR-NK cells under four different antigen combinations. The right-top panel summarizes the four core functional breakthroughs of the aCAR-iCAR system, including on-target/off-tumor toxicity blockade, inhibition of trogocytosis-mediated effector cell fratricide and antigen escape, reversal of TME-mediated NK cell functional exhaustion, and mitigation of immune synapse impairment caused by ICAM-1 downregulation on tumor cells. The bottom-right panel presents the AI-optimized three-level safety switch system, including the physiological safety switch mediated by iCAR, the pH-responsive nanocarrier-based drug-responsive safety switch, and the inducible depletion-based suicide switch, to achieve full-range toxicity control of the dual-module CAR-NK therapy. CAR, chimeric antigen receptor; aCAR, activating CAR; iCAR, inhibitory CAR; NK, natural killer; TME, tumor microenvironment; ITIM, immunoreceptor tyrosine-based inhibitory motif; scFv, single-chain fragment variable; TGF-β, transforming growth factor-β; IL-10, interleukin-10; ICAM-1, intercellular adhesion molecule 1.

The structure and function of the aCAR-iCAR system are shown in **Figure 2**.

### 3.2 Synergistic optimization directions of the dual-module CAR system

The ubiquitous antigen heterogeneity of solid tumors and insufficient *in vivo* persistence of effector cells may be effectively addressed via synthetic biology engineering. When combined with AI-empowered rational optimization, this approach may also help mitigate potential risks including signal leakage and excessive cell activation. All optimization strategies described in this section remain at the conceptual or early preclinical stages (Level 2–3 evidence).

#### 3.2.1 Multispecific CAR structure fusion

The integrated design of tandem multispecific CARs with the dual-module system is predicted to reduce the tumor antigen escape rate by more than 60% (53). This strategy is classified as Level 2 evidence: multispecific CAR designs have been validated in CAR-T systems, while their application in dual-module CAR-NK cells remains at the proof-of-concept stage. The antibody structure optimization algorithm AbLIFT (antibody light-heavy chain interface optimization tool) may support automated optimization of the scFv structures of both aCAR and iCAR, reduce molecular steric hindrance, decrease non-specific binding, and better accommodate the synergistic action mode of dual scFvs (54). AI-driven CAR structure optimization strategies are further elaborated in Chapter 4.

#### 3.2.2 Integration of Cis-acting functional elements and synergistic engineering strategies

Integration of proteolysis-targeting chimera (PROTAC) degradation modules, cytokine engineering cassettes (e.g., membrane-bound or secretory IL-15/IL-21), and hypoxia-adaptive metabolic reprogramming elements substantially enhances dysfunction resistance and *in vivo* persistence of CAR-NK cells (23, 55), aligning with the TME-driven dysfunction mechanisms detailed in Section 3.1.2.3. These strategies are collectively classified as Level 2 evidence: most have been functionally validated in CAR-T or conventional CAR-NK systems, while their synergistic application with dual-module CAR designs remains at the preclinical exploratory stage.

AI-mediated co-design of lactate transporters and the dual-module CAR is proposed to enable CAR-NK cells to utilize lactate and acetate in the TME for energy supply, alleviate metabolic suppression, and achieve a precise balance between tumoricidal function and *in vivo* persistence (56). This specific metabolic engineering strategy falls under Level 2–3 evidence, with core supporting data primarily derived from CAR-T metabolic reprogramming studies.

Beyond these molecular modules, a broader array of NK cell engineering strategies has well-documented preclinical efficacy and can be synergistically coupled with the dual-module CAR system to further enhance antitumor efficacy against solid tumors (Level 2 evidence).

CISH knockout eliminates the intracellular negative regulator of cytokine signaling, markedly amplifying responsiveness to IL-15 and enhancing both the *in vivo* persistence and antitumor cytotoxicity of NK cells (57).

TGFBR2 editing abrogates TGF-β-mediated inhibitory signal transduction and directly counteracts TME-driven metabolic suppression and functional exhaustion, which is particularly relevant for the immunosuppressive subtype (see **Table 1**, Type II) (23, 58).

**Table 1:** Proposed four-subtype precision therapeutic strategy of aCAR-iCAR dual-module CAR-NK therapy for solid tumors.

Subtype	Subtype name	Core molecular features	Dominant resistance mechanism	Recommended therapeutic strategy	Representative cancer types
Type I	HLA-I LOH type	Classical HLA-I (A/B/C) loss; variable HLA-E; intact ICAM-1; low stroma (121, 122)	Trogocytosis-induced antigen loss & fratricide (14); mainly evades T-cell immunity	KIR-based iCAR + tumor-specific aCAR; iCAR not engaged by HLA-I-low tumors, enabling unopposed aCAR-mediated killing (14)	Melanoma, NSCLC (123)
Type II	Immunosuppressive type	High HLA-E; enriched TGF-β/IL-10; upregulated NKG2A on tumor-infiltrating NK cells (26)	TME-driven exhaustion via persistent NKG2A-HLA-E signaling; mainly suppresses NK-cell immunity (124)	Potent aCAR + NKG2A-iCAR; combined with NKG2A monoclonal antibody (125)	Pancreatic cancer, HCC (126)
Type III	Adhesion defect type	Low/absent ICAM-1; normal other adhesion molecules (50)	Impaired synapse formation from ICAM-1 downregulation (50); mainly impairs NK-cell ADCC	NK-adapted aCAR with strong activating signals; ICAM-1-independent synapse formation (50, 127)	Glioblastoma, ovarian cancer (106)
Type IV	Matrix-rich type	Dense ECM; severe chemokine mismatch; high stromal content (116)	Insufficient infiltration + profound TME immunosuppression (128); physical barriers & chemokine traps	aCAR-iCAR + AI-optimized stroma-degrading nanodelivery; multiplexed targeting of tumor cells & CAFs (119)	Gastric cancer, CRC (85)

This four-subtype classification is a theoretical framework based on mechanistic reasoning and retrospective correlative evidence, and requires prospective clinical validation. Biomarker cut-offs for subtype classification have not been standardized and may vary across tumor types. The dominant resistance mechanism may vary within each subtype depending on additional tumor-intrinsic and microenvironmental factors. All recommended therapeutic strategies are supported by preclinical evidence with varying validation levels, as detailed in **Table 2**. See Section 6.1 for detailed clinical rationale, detection approaches, and biomarker assessment criteria.

aCAR, activating chimeric antigen receptor; ADCC, antibody-dependent cell-mediated cytotoxicity; CAFs, cancer-associated fibroblasts; CAR-NK, chimeric antigen receptor-natural killer; CRC, colorectal cancer; ECM, extracellular matrix; HLA, human leukocyte antigen; HCC, hepatocellular carcinoma; ICAM-1, intercellular adhesion molecule 1; iCAR, inhibitory chimeric antigen receptor; IL-10, interleukin-10; LOH, loss of heterozygosity; NK, natural killer; NSCLC, non-small cell lung cancer; TGF-β, transforming growth factor-β; TME, tumor microenvironment.

Chemokine receptor engineering (e.g., CXCR1/CXCR2 overexpression) improves NK cell tumor homing by matching chemokine gradients in the TME and addresses the infiltration barrier in matrix-rich subtypes (see **Table 1**, Type IV) (49).

Memory-like NK cell induction via pre-activation with IL-12/15/18 endows cells with enhanced cytotoxicity and long-term survival capacity, which can be integrated with the dual-module CAR design to further boost therapeutic durability (42).

induced pluripotent stem cell-derived natural killer (iPSC-NK) platforms inherently provide a genetically uniform, scalable cell source, offering a potential solution to the manufacturing bottlenecks and inter-donor variability that limit primary NK cell products.

Armored CAR-NK designs co-expressing immunostimulatory cytokines (e.g., IL-12, IL-15, IL-18) or co-stimulatory ligands (e.g., 4-1BBL) further enhance effector function and remodel the TME, and are conceptually compatible with dual-module CAR architectures (55).

In addition to cell-intrinsic engineering, combinatorial strategies represent a critical translational direction for amplifying therapeutic efficacy. Combination with immune checkpoint blockade, radiotherapy, oncolytic viruses, stromal remodeling agents, or NK cell engagers — bispecific antibodies that crosslink NK cell-activating receptors with tumor surface antigens to recruit and activate endogenous NK cells — can remodel the immunosuppressive TME, degrade physical stromal barriers, and recruit endogenous immune effector cells to create a more permissive microenvironment for dual-module CAR-NK function. These combinations are particularly relevant for overcoming the TME-mediated physical and immune barriers described in Section 2.1 (26, 30, 59).

The above cell-intrinsic engineering strategies and combinatorial therapeutic approaches, together with the dual-module CAR system, form a comprehensive functional regulatory framework. Multi-parameter global optimization of this framework cannot be efficiently accomplished via traditional trial-and-error approaches. Leveraging the inherent advantages of high-throughput rational design, artificial intelligence offers a promising solution to this technical challenge, with relevant design systems and application strategies systematically elaborated in Chapter 4.

## 4 AI-empowered end-to-end rational design framework for dual-module CARs

Maximizing the functional efficacy of the aCAR-iCAR system depends on multi-dimensional global optimization, yet the conventional univariate trial-and-error approach is insufficient to meet the optimization demands of millions of parameter combinations inherent to the system. In this chapter, we propose an AI-empowered end-to-end design framework that comprehensively covers the entire workflow of target screening, structural design, signal regulation, and clinical translation, supporting the advancement of the CAR-NK R&D paradigm from “empirical trial-and-error” to “rational design”. All AI-based strategies described in this chapter are stratified by evidence level, consistent with the grading system outlined in Chapter 3.

### 4.1 High-throughput precise screening of target combinations

The target combination of aCAR and iCAR is a key determinant of the breadth of the therapeutic window and potential clinical benefit (60), consistent with the off-target toxicity regulatory mechanisms detailed in Section 3.1.2.1. AI technologies based on multi-omics data and deep learning algorithms may support high-throughput, context-specific precision target screening (61). This category of AI screening tools is generally classified as Level 2 evidence, with robust validation in pan-cancer target discovery settings. Graph neural networks (GNNs) and Transformer models (62) can be applied to integrate multi-source public databases including The Cancer Genome Atlas (TCGA), Genotype-Tissue Expression (GTEx), and the Human Tumor Atlas Network (HTAN) (63), to address three core screening tasks: 1) screening of tumor-specific aCAR target antigens and evaluation of their spatial heterogeneity (60); 2) prediction of antigen loss and clonal evolutionary trajectories to screen target combinations with low immune escape risk; and 3) analysis of target expression compatibility to reduce the risk of normal tissue co-expression and off-target toxicity (63).

AI technologies may help address multiple key pain points in CAR-NK clinical translation, as outlined in Section 3.1.4. The GETgene AI framework proposed by Devhare et al. offers a promising paradigm for the precise identification of potential therapeutic targets via the integration of multi-omics data and AI algorithms (64). On this basis, machine learning algorithms (such as DASH) have been validated to detect HLA LOH from sequencing data with high sensitivity, representing a valuable tool for the precise screening of populations with clinical benefit (65). Integration of ICAM-1 and HLA-E expression profiles supports the construction of a standardized patient stratification system (50). Integrated multi-omics analysis may help characterize the metabolic heterogeneity of the TME and identify key metabolic regulatory targets (66). In addition, the BayesTS algorithm can be used to quantify antigen binding specificity, offering a quantitative reference standard for target screening (67); AI-based approaches may enable simultaneous screening of targets in tumor cells and cancer-associated fibroblasts (CAFs) (68).

### 4.2 AI-driven CAR structure design and optimization

Breakthrough advances in AlphaFold3 and the development of diffusion generative models have spurred the exploration of generative AI in CAR structure design, supporting the development of *de novo* rational design strategies for CAR molecules (69, 70). This category of structural AI tools is classified as Level 2 evidence, with robust validation in general protein structure prediction, while their dedicated application in dual-module CAR-NK design remains at an early exploratory stage. A suite of dedicated AI models has shown promising application potential for structural optimization of dual-module CAR systems (53), aligning with the multispecific CAR fusion design strategies detailed in Section 3.2.1. Among these, the CAR-Toner platform leverages artificial intelligence to predict CAR tonic signaling (Level 2 evidence, validated in CAR-T systems), offering a promising reference strategy for the rational optimization of dual-module CAR architectures (71). AI-driven molecular docking approaches support computational screening of scFv candidates for anti-CD30 CARs, with binding specificity requiring further experimental validation (72). The AGILE platform accelerates lipid nanoparticle (LNP) development via deep learning, offering an efficient research tool for mRNA delivery into primary NK cells (73). AI-optimized LNP delivery strategies are further elaborated in the AI-nanosymbiont framework in Chapter 5.

Furthermore, artificial intelligence is proposed to support global synergistic optimization of core components within CAR molecules (Level 2–3 evidence for dual-module CAR applications), including scFv affinity and stability, structural compatibility of transmembrane and hinge regions, combinations of NK-specific signaling domains, and the dimerization properties of NKG2A-based iCARs (53). This strategy directly addresses the parameter optimization challenges of dual-module CAR systems outlined in Section 3.1.4. Machine learning-mediated screening of synthetic signaling domains supports the identification of novel signaling motifs that combine potent cytotoxicity with stem-like properties, offering a potential solution to the technical bottlenecks of conventional experimental approaches in optimizing combinatorial parameters (74).

### 4.3 AI-driven signal balance and dynamic calibration of the aCAR-iCAR system

The dynamic balance between aCAR activating signals and iCAR inhibitory signals is a core determinant of the potential clinical therapeutic efficacy of dual-module CAR-NK cells (75), consistent with the AND-NOT Boolean logic gate regulatory principle detailed in Section 3.1.1. Interpretable graph neural network (GNN) models (Level 2 evidence), which integrate multi-dimensional molecular features and machine learning approaches, support the precise global dissection of signaling networks and the prediction of regulatory outcomes, and help systematically unravel the cross-regulatory mechanisms between signaling pathways (76). The complexity of NK cell inhibitory signaling is underscored by the involvement of multiple regulatory components, including adaptor proteins such as β-arrestin 2 that interact with SHP-1/SHP-2 phosphatases to modulate inhibitory signal transduction (77).

These models integrate molecular signaling data and clinical sample datasets to characterize three core regulatory loci: (1) The ITIM locus of KIR2DL1 governs the inhibitory strength of KIR-based iCARs and suppresses NK cell cytotoxicity via recruitment of SHP-1/SHP-2 phosphatases (51); (2) The ITIM motif of NKG2A provides the structural basis for dynamic regulation of inhibitory signals in NKG2A-based iCARs, which are transduced upon binding to HLA-E peptides (26); (3) The immunoreceptor tyrosine-based activation motif (ITAM) of CD3ζ defines the activation threshold of aCAR molecules (78).

AI may support the screening of optimal signal balance schemes via global parameter scanning (Level 2–3 evidence for dual-module CAR applications). This approach directly addresses the multi-parameter optimization challenge outlined in Section 3.1.4. Notably, clinical data from conventional CAR-NK therapies provide a reference for signal balance optimization: clinical studies have shown that in patients with B-cell malignancies, treatment with optimized donor-derived CD19-specific CAR-NK cells achieved a 1-year overall survival rate of 94% (75). This body of clinical evidence is classified as Level 2, supporting the rationale for signaling optimization in dual-module CAR-NK design. In leukemia mouse models, pluripotent stem cell-derived CAR-iNK cells exhibited long-term persistence for over 80 days and effectively suppressed tumor recurrence (79), providing preclinical reference evidence for signaling optimization strategies. In solid tumor models, key targets identified via genome-wide CRISPR screening significantly enhanced the antitumor efficacy of CAR-NK cells (57), offering candidate loci for the functional optimization of dual-module systems. Furthermore, integration of single-cell perturbation screening and machine learning technologies may facilitate the elucidation of the cross-regulatory network between CAR signaling and the innate pathways of NK cells. Studies have constructed a pan-cancer, multi-dataset single-cell transcriptional reference atlas of human NK cells, which integrates transcriptomes of tumor-infiltrating NK cells from 39 datasets and 427 patients, and characterizes six functionally distinct NK cell states (80). This atlas provides a foundational resource for dissecting TME-mediated NK cell dysfunction, as discussed in Section 2.1. Subsequent integration of gene regulatory network analysis with artificial intelligence supports the prediction of immune cell state transitions and patient prognosis (81).

### 4.4 AI-driven clinical decision optimization: from patient stratification to digital twins

AI multimodal models (Level 2–3 evidence for dual-module CAR-NK applications) may support the integration of multi-dimensional clinical data to provide potential decision-making frameworks for core clinical scenarios, including patient stratification, personalized dosing regimen design, and toxicity prevention and management (82–84), aligning with the three-tier safety switch system described in Section 3.1.3 and addressing the clinical translation challenges outlined in Section 3.1.4. Studies have demonstrated that AI clinical prediction models may help predict patient’ treatment response rates, *in vivo* persistence of CAR-NK cells, and toxicity risk. Most supporting data are derived from conventional CAR-NK or CAR-T clinical settings, serving as a reference for dual-module CAR-NK clinical development. The patient stratification system constructed based on the expression profiles of HLA-I/HLA-E/ICAM-1 supports precision patient selection for dual-module CAR-NK therapy (85), consistent with the target screening and patient stratification strategies detailed in Section 4.1.

For dosing decision-making, population pharmacokinetics/pharmacodynamics (PK/PD) models integrate preclinical pharmacokinetic data and human tumor data to inform optimal dosing regimen selection for pancreatic cancer clinical trials. This approach is classified as Level 2 evidence, with established application in conventional CAR-T and oncology clinical trials. In pancreatic cancer immunotherapy, patients screened via AI-assisted multi-dimensional data integration achieved an objective response rate of 33.3% and a disease control rate of 90.3%, offering a quantitative reference for identifying populations that may derive clinical benefit from dual-module CAR-NK therapy (86).

In addition, tumor digital twin technology supports in silico exploratory validation of treatment regimens, may help predict therapeutic efficacy and adverse events in advance, and may reduce clinical translation risks and R&D costs. The DITTO digital twin system, via deep reinforcement learning, has been validated to predict tumor control efficacy as well as short- and long-term toxicity risks in patients with head and neck cancer (Level 2 evidence, validated in solid tumor standard-of-care settings), providing a methodological reference for the clinical development of dual-module CAR-NK regimens after fully weighing the benefit-risk ratio (87). Digital twin-based regimen validation complements the safety switch framework described in Section 3.1.3.

### 4.5 Data, model, and interpretability: three core challenges in AI-driven CAR-NK design

Currently, the technical framework of AI-driven dual-module CAR-NK design still faces three core technical bottlenecks at the fundamental level.

First, AI models rely heavily on large-scale, high-quality standardized datasets, yet the paucity of high-quality dual-module CAR-NK-specific data may restrict the generalization performance and cross-cancer transferability of such models (88, 89).

Second, existing computational models remain unable to fully recapitulate the non-linear intercellular interactions within the TME, which may lead to discrepancies between in silico efficacy predictions and actual *in vivo* outcomes (90).

Third, the “black box” nature of deep learning models renders the causal chain of molecular design decisions opaque, which poses challenges to mechanistic interpretation and targeted iterative optimization of CAR structures (91).

In summary, this chapter systematically outlines the AI-empowered rational design framework for endogenous structural and signaling optimization of dual-module CAR-NK cells. However, overcoming the physical stromal and *in vivo* delivery barriers of solid tumors may benefit from the further integration of exogenous nanodelivery technologies, which are the core focus of Chapter 5.

## 5 AI-nanosymbiont: the exogenous synergistic system for clinical translation of dual-module CAR-NK

Building on the endogenous optimization framework detailed in Chapter 4, this chapter systematically investigates the application strategies of AI-nanosymbiont technology for dual-module CAR-NK therapy.

In this review, the term “AI-nanosymbiont” is explicitly defined as an integrated synergistic system coupling AI-optimized nanodelivery technologies with dual-module CAR-NK cellular engineering. The “symbiont” designation underscores that the interaction between exogenous nanomaterials and endogenous immune cell engineering is a mutually potentiating relationship, rather than a mere superposition of two independent technologies.

All nanotechnology-based strategies described in this chapter are classified as Level 2–3 evidence, with core validation in conventional CAR-NK systems, while their combination with dual-module designs remains at the preclinical exploratory stage.

### 5.1 AI-driven mRNA-LNP delivery platform and *in vivo in situ* CAR-NK engineering

AI-optimized nanocarriers may help address key challenges including low mRNA transfection efficiency and heterogeneous aCAR/iCAR expression, support *in vivo in situ* dual-module CAR-NK engineering, and alleviate the scalability bottleneck of *in vitro* manufacturing (73), aligning with the manufacturing and translational challenges outlined in Section 3.1.4.

Conventional viral delivery vectors carry inherent limitations, including lengthy manufacturing cycles, high production costs, and the risk of insertional mutagenesis. Lipid nanoparticles (LNPs), as a core class of non-viral delivery vectors, offer distinct advantages for clinical translation (92–94). The AGILE AI platform, via the synergistic combination of deep learning and combinatorial chemistry, has characterized the cell-type-specific tropism of ionizable lipids, providing important reference for the optimization of NK cell-targeted delivery in dual-module CAR systems (73). This finding is classified as Level 2 evidence. Studies have demonstrated that CD19-CAR and BCMA-CAR mRNA-LNPs achieved 78% and 95% CAR expression efficiency in NK cells, respectively, exhibiting robust mRNA transfection efficiency (95). These data are derived from conventional single-target CAR-NK systems (Level 2 evidence), serving as a foundational reference for dual-module CAR-NK delivery development. Furthermore, the CD56-targeted NK cell-specific LNP strategy may support *in vivo in situ* CAR-NK engineering, which may substantially reduce the manufacturing cycle of conventional *in vitro* manufacturing workflows (96). Notably, this strategy is still at the preclinical proof-of-concept stage, and its *in vivo* targeting efficiency and long-term safety profiles remain to be further validated. Safety evaluation of *in situ*-engineered CAR-NK cells aligns with the three-tier safety switch principle defined in this work.

### 5.2 Nanotechnology-driven TME remodeling and synergistic potentiation of CAR-NK cells

In solid tumors, impaired intratumoral infiltration of dual-module CAR-NK cells represents a key bottleneck restricting their therapeutic efficacy, consistent with the stromal physical barrier and TME-mediated dysfunction described in Section 2.1. The AI-nanosymbiont may support multi-dimensional synergistic potentiation via precise remodeling of the TME (35, 97, 98). This integrated AI-nanosymbiont strategy is classified as Level 2–3 evidence, with core mechanistic validation in conventional CAR-NK systems.

Multiple nanotherapeutic strategies may support synergistic TME remodeling (Level 2 evidence for foundational efficacy): matrix metalloproteinase (MMP)-responsive nanocarriers may enhance the infiltration of dual-module CAR-NK cells into the tumor core region by degrading the extracellular matrix (ECM) (59); acidic microenvironment-responsive nanocarriers may deliver TGF-β inhibitors to alleviate immunosuppression and help restore the antitumor activity of NK cells (58); additionally, demethylating agents may upregulate ICAM-1 expression on tumor cells and increase their susceptibility to NK cell-mediated cytotoxicity (99), complementing the ICAM-1-based patient stratification strategy detailed in Section 4.1.

Furthermore, nanoparticle backpack technology (Level 2 evidence) may support conjugation of cytokines such as interleukin-15 (IL-15) to the surface of immune cells, enabling controlled *in situ* release of cytokines upon CAR activation. This strategy may substantially enhance cellular proliferative capacity while reducing the risk of systemic cytokine toxicity, and help prolong *in vivo* persistence and augment the antitumor activity of dual-module CAR-NK cells (100–102), aligning with the persistence optimization strategies discussed in Section 3.2.2.

### 5.3 Non-invasive *in vivo* tracking and precise toxicity management

The AI-nanosymbiont may support non-invasive *in vivo* tracking of dual-module CAR-NK cells and multi-dimensional precision toxicity management (Level 2–3 evidence for dual-module applications). Specifically, labeling dual-module CAR-NK cells with nanoscale imaging probes, combined with magnetic resonance imaging (MRI) or positron emission tomography/computed tomography (PET/CT), supports non-invasive whole-body real-time monitoring (103). This imaging-based tracking strategy is classified as Level 2 evidence, with robust preclinical validation in conventional CAR-NK systems. On this basis, AI imaging models may support quantitative evaluation of therapeutic efficacy, prediction of recurrence risk, and iterative improvement of *in vivo* efficacy prediction accuracy (104), complementing the clinical efficacy prediction framework outlined in Section 4.4. Meanwhile, acidic microenvironment-responsive nanocarriers may deliver regulatory molecules to selectively activate therapeutic functions within the acidic tumor pH microenvironment, while reducing systemic adverse effects and supporting individualized safety management (105).

This synergistic system integrating endogenous cellular engineering and exogenous nanodelivery offers a comprehensive technical framework to support the clinical translation and individualized application of dual-module CAR-NK therapy, addressing the core translational challenges outlined in Section 3.1.4.

**Figure 3:**
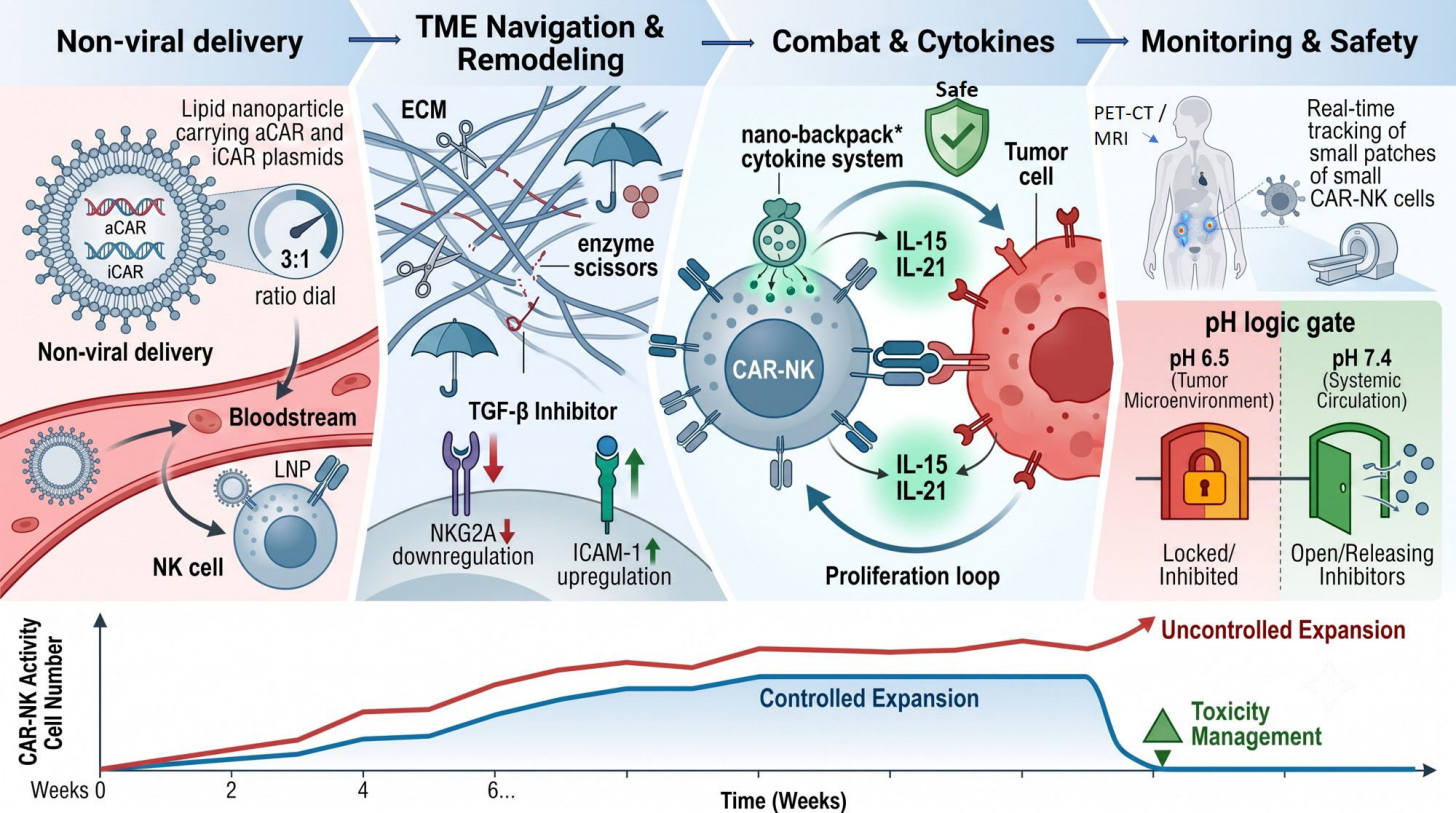
AI-nanosymbiont synergistic system empowering dual-module CAR-NK therapy in solid tumors. This schematic illustrates how an AI-optimized nanotechnology platform synergizes with activating/inhibitory (aCAR/iCAR) dual-module CAR-NK cells to overcome three major barriers: *in vivo* delivery, tumor microenvironment (TME) immunosuppression, and clinical safety control. Panel 1 (Non-viral delivery): AI-optimized lipid nanoparticles (LNPs) enable non-viral delivery of aCAR and iCAR mRNA with precise ratio control, supporting *in vivo in situ* CAR-NK engineering. Panel 2 (TME Navigation & Remodeling): The nano-system remodels the TME by degrading extracellular matrix (ECM) barriers, delivering transforming growth factor-β (TGF-β) inhibitors, downregulating NKG2A, and upregulating intercellular adhesion molecule 1 (ICAM-1), to enhance NK cell tumor infiltration. Panel 3 (Combat & Cytokines): A nano-backpack cytokine system releases IL-15/IL-21 upon CAR activation, driving localized CAR-NK proliferation and persistence without systemic cytokine toxicity. Panel 4 (Monitoring & Safety): CAR-NK cells are tracked in real time via MRI/PET-CT imaging. A pH-responsive nanocarrier safety switch remains locked at acidic tumor pH (6.5) to preserve on-target activity, and opens at physiological pH (7.4, systemic circulation) to release inhibitors for rapid toxicity management. Bottom panel (Kinetic profile): The timeline graph contrasts uncontrolled CAR-NK expansion (red, associated with high toxicity risk) with the controlled expansion and precise toxicity management enabled by this synergistic platform (blue). CAR, chimeric antigen receptor; aCAR, activating CAR; iCAR, inhibitory CAR; NK, natural killer; AI, artificial intelligence; TME, tumor microenvironment; LNPs, lipid nanoparticles; mRNA, messenger RNA; ECM, extracellular matrix; TGF-β, transforming growth factor-β; ICAM-1, intercellular adhesion molecule 1; NKG2A, natural killer group 2A; IL, interleukin; MRI, magnetic resonance imaging; PET-CT, positron emission tomography-computed tomography.

The synergistic mechanism of the AI-nanosymbiont system is illustrated in **Figure 3**.

## 6 Current status of clinical translation, core challenges, and future research and development roadmap

The preceding chapters have systematically outlined the AI-empowered endogenous structural design and signaling regulation strategies for dual-module CAR-NK cells, as well as nanotechnology-mediated exogenous TME remodeling and *in vivo* delivery solutions. Building on this framework, this chapter systematically reviews the current clinical translation landscape of CAR-NK therapies, summarizes global clinical trial progress, proposes a four-subtype precision treatment paradigm based on the molecular heterogeneity and cascaded drug resistance features of solid tumors, and analyzes core translational bottlenecks and future R&D roadmaps to provide theoretical and practical reference for the clinical advancement of this technology.

### 6.1 Current status of clinical translation and the four-subtype precision treatment strategy for dual-module CAR-NK

Although conventional CAR-NK therapy has demonstrated well-documented efficacy and a favorable safety profile in hematologic malignancies, its clinical benefit in solid tumors remains limited. A primary contributor to this translational dilemma is the vicious cycle of cascading pathological resistance mechanisms detailed in Chapter 2.

While both dual-module CAR and AI-empowered CAR technologies remain at the preclinical research stage (Level 2–3 evidence), their antitumor activity has been preliminarily validated across multiple solid tumor models (95). Conventional multispecific CAR-NK products have advanced to phase I clinical trials (Level 2 clinical evidence) (106), and AI-optimized LNP-mediated *in vivo in situ* engineering technology is regarded as an early-stage estimate for clinical trial initiation in the coming years, providing a potential translational reference for dual-module CAR-NK development. As supporting clinical translational evidence, the phase II trial of the NKG2A monoclonal antibody monalizumab plus cetuximab achieved an objective response rate (ORR) of 31% in recurrent/metastatic head and neck squamous cell carcinoma (HNSCC), providing early clinical proof-of-concept for the NKG2A-HLA-E axis targeting strategy and iCAR component design in dual-module CAR systems (107). However, the final 2025 results of the phase III INTERLINK-1 clinical trial (NCT04590963) confirmed that this combination regimen did not significantly improve overall survival (OS) in patients (median OS 8.8 months vs. 8.6 months, HR = 0.97, P = 0.72). This negative result highlights the inherent limitations of systemic NKG2A checkpoint monotherapy, and requires clear differentiation from the NKG2A-based iCAR design proposed in this review. Three core mechanistic differences explain why the antibody trial failure does not negate the translational value of iCAR strategies:

First, opposite modes of action: anti-NKG2A antibodies work as systemic activators that non-selectively block inhibitory signals on all circulating NK and T cells, yet fail to provide sufficient tumor-localized activation to overcome the profoundly immunosuppressive solid tumor TME. This systemic blockade, divorced from spatial control, can also trigger off-target inflammatory effects. By contrast, NKG2A-iCARs act as a tumor-selective safety brake: they only deliver inhibitory signals when binding HLA-E on normal tissues, and do not interfere with aCAR-driven tumor killing in HLA-E-low tumor regions. This spatially regulated logic avoids the systemic immune overdrive that limits antibody efficacy.

Second, different responses to antigen heterogeneity: monalizumab’s failure is partly driven by heterogeneous HLA-E expression across tumor clones, which leaves some tumor cells unresponsive to checkpoint blockade. For iCAR systems, HLA-E heterogeneity does not weaken antitumor efficacy — it only reduces inhibitory signaling in HLA-E-deficient tumor clones, thereby selectively permitting aCAR-mediated killing of those clones, which is fully consistent with NK cells’ native “missing self” activation principle.

Third, distinct activating signal backgrounds: anti-NKG2A antibodies rely entirely on endogenous NK cell activating signals to work, and these signals are often suppressed in the solid tumor TME. NKG2A-iCARs are paired with a robust aCAR activating module that provides a synthetic activating signal independent of endogenous receptors, fundamentally overcoming the weak activation tone that limits checkpoint monotherapy (108).

Taken together, the INTERLINK-1 result shows that isolated systemic NKG2A blockade is insufficient for solid tumors, and in turn supports the rationale for engineered dual-module CAR systems that combine tumor-selective inhibition with synthetic activating signals.

Based on the molecular expression profiles of solid tumors and the cascading drug resistance mechanisms outlined in the preceding chapters, this review proposes a four-subtype precision treatment paradigm to support the rational matching of tumor molecular features, resistance mechanisms, and therapeutic regimens.

As a proposed clinical workflow yet to be prospectively validated, each subtype corresponds to standardized detection approaches: Type I (HLA-I LOH type) is identified via next-generation sequencing-based HLA typing and LOH analysis; Type II (immunosuppressive type) is evaluated by immunohistochemistry for HLA-E and TGF-β expression; Type III (adhesion defect type) is determined via ICAM-1 immunohistochemical quantification; Type IV (matrix-rich type) is assessed via radiological stromal density scoring or transcriptomic stromal signature analysis.

The specific subtypes and corresponding strategies are summarized in **Table 1**, aligning with the patient stratification framework outlined in Section 4.1.

### 6.2 Core bottlenecks and challenges in clinical translation

The clinical translation of dual-module CAR-NK therapies faces core bottlenecks across three dimensions: manufacturing technology, system compatibility, and regulatory compliance, consistent with the overarching translational challenges outlined in Section 3.1.4.

#### 6.2.1 Manufacturing and quality control bottlenecks of clinical-grade CAR-NK cells

The high structural complexity of the dual-module CAR poses inherent challenges to achieving balanced, stable expression of aCAR and iCAR in primary NK cells, which may lead to imbalanced stoichiometry between the two modules (14). Furthermore, NKG2A-based iCARs are prone to non-specific dimerization with endogenous CD94, which may trigger aberrant signal transduction; scFv fragments are susceptible to *in vivo* degradation, leading to a progressive decline in CAR surface expression levels, which in turn impairs long-term antitumor activity (109).

For the development of off-the-shelf cell products, inter-donor variability, variable differentiation efficiency of iPSC-NK cells, tumorigenicity risk, and the lack of unified industry-wide quality control standards collectively represent key technical barriers to standardized clinical translation (110, 111). These manufacturing challenges are classified as Level 2 translational barriers, with well-documented impact on conventional CAR-NK product development.

#### 6.2.2 Compatibility challenges of AI-empowered technologies in clinical translation

When translated from computational design to clinical scenarios, the aforementioned technical limitations of AI frameworks (detailed in Section 4.5) are further amplified into three core compatibility challenges.

First, the scarcity of high-quality clinically annotated datasets may substantially limit the generalization performance of models in real-world patient populations, leading to greater discrepancies between predicted and actual therapeutic outcomes (112).

Second, current static AND-NOT Boolean logic gates cannot fully adapt to spatiotemporal antigen heterogeneity and dynamic tumor clonal evolution within the TME, which may lead to aberrant signal recognition and subsequent therapeutic failure (60).

Third, the limited traceability of AI-driven design decisions poses practical challenges to clinical accountability and adverse event attribution, which fails to meet the full-lifecycle management requirements for clinical-grade therapeutic products, aligning with the safety control framework outlined in Section 3.1.3.

#### 6.2.3 Gaps in the regulatory framework and evaluation system

Beyond manufacturing and technical compatibility hurdles, the clinical translation of this therapy is further constrained by the absence of dedicated global regulatory guidelines and undefined core evaluation metrics such as immunogenicity assessment and long-term safety follow-up (111, 113). From a product safety perspective, several key risks require specific regulatory consideration.

First, for off-the-shelf iPSC-derived NK products, tumorigenicity risk from residual undifferentiated pluripotent cells represents a core safety concern, requiring standardized *in vivo* tumorigenicity assays (e.g., subcutaneous or intravenous administration in immunodeficient mice) and genomic stability testing (e.g., karyotyping, whole-genome sequencing for copy-number alterations) prior to clinical use. Second, gene editing procedures (e.g., CRISPR-Cas9-mediated endogenous receptor knockout) carry off-target editing risks that may induce aberrant cell function or malignant transformation, necessitating comprehensive off-target analyses (e.g., GUIDE-seq, CIRCLE-seq, or whole-genome sequencing) in quality control workflows. Third, non-viral LNP delivery systems may induce acute infusion-related reactions and innate immune activation (e.g., complement activation-related pseudoallergy and TLR-mediated inflammatory responses), and their *in vivo* biodistribution and long-term metabolic fate in human subjects remain insufficiently characterized. Fourth, allogeneic NK cell products face potential risks of host immune rejection, which can limit their *in vivo* persistence and therapeutic efficacy. While NK cells inherently carry a substantially lower risk of graft-versus-host disease (GVHD) than T cells, host-versus-graft alloreactivity and pre-existing anti-HLA antibodies in sensitized patients remain important translational considerations. As with all genetically engineered cell products, long-term follow-up is required to monitor potential late adverse events, with the duration adapted to the genomic integration status of the engineering approach.

For AI-assisted therapeutic products, additional requirements for algorithm transparency, result reproducibility, and post-marketing pharmacovigilance are expected to be incorporated into future regulatory frameworks, directly addressing the interpretability gap of deep learning models outlined in Section 4.5. Meanwhile, as an emerging therapeutic modality, *in vivo in situ* cell engineering lacks clearly defined regulatory classification and approval criteria, representing a key non-technical barrier to its clinical translation (114), consistent with the *in situ* engineering technologies described in Section 5.1.

Beyond product-specific risks, additional translational factors warrant integrated regulatory consideration. These include the limited *in vivo* persistence of mRNA-LNP-transfected CAR-NK cells (consistent with the transient expression characteristics outlined in Section 5.1), the need for cytokine support strategies to sustain effector function (consistent with the nanoparticle backpack delivery system described in Section 5.2), and the immunogenicity risks of repeated LNP administration. For viral vector-based manufacturing approaches, insertional mutagenesis and clonal expansion risks remain key safety endpoints requiring long-term surveillance. A comprehensive regulatory framework for dual-module CAR-NK products will need to address all of these dimensions to support safe and standardized clinical translation.

### 6.3 Future R&D roadmap

To address the aforementioned translational bottlenecks outlined in Section 6.2, future research efforts should integrate multidisciplinary technologies to pursue core strategic goals of precision, manufacturability, scalability, and accessibility. Key actionable R&D directions are proposed as follows:

#### 6.3.1 AI-driven spatiotemporally adaptive dual-module CAR systems

To overcome the limitation of static AND-NOT Boolean logic gates in adapting to intratumoral antigen heterogeneity, future designs are proposed to integrate spatial transcriptomics and tumor clonal evolution prediction models to construct ternary dynamic CAR architectures, with fine-tuning of intracellular signaling strength as a core optimization dimension (115). In parallel, PROTAC degradation modules, metabolic reprogramming cassettes and extracellular matrix stiffness-targeting strategies are proposed to be coupled to establish standardized combinatorial engineering regimens, further enhancing CAR-NK cell resistance to TME-mediated suppression (59, 116).

#### 6.3.2 Closed-loop R&D system: AI design–organoid validation–clinical translation

This direction establishes a three-module closed-loop validation pipeline to bridge preclinical findings and clinical outcomes: first, patient-derived tumor organoid models are applied for high-fidelity functional validation of dual-module CAR-NK cells; second, interpretable AI tools are integrated to improve the traceability of design decisions and address the “black box” limitation of deep learning; third, tumor digital twin technology is incorporated to simulate efficacy and toxicity in silico, reducing translational risk and R&D cost. This multi-link closed R&D paradigm has been widely recognized as a core path for the next generation of CAR-NK technology translation in the latest field consensus reviews (117).

#### 6.3.3 Scalable manufacturing technologies for off-the-shelf products

Optimization of iPSC-NK directed differentiation and precise gene editing systems is prioritized as the foundation of off-the-shelf product manufacturing, to improve the expression homogeneity of dual-module CARs and reduce the risk of aberrant signal leakage. In parallel, functionalized lipid nanoparticle-based non-viral delivery systems have shown robust potential in enabling efficient chemical and genetic fortification of NK cells, representing a key technical direction for standardized and large-scale manufacturing of CAR-NK products (118). On this basis, novel persistent CAR expression platforms such as circRNA-based constructs are further explored to reduce antigen shedding and prolong the functional duration of engineered cells (109).

#### 6.3.4 Translation of personalized therapeutic strategies

AI-based multi-omics analysis is proposed to customize individual target combinations and signaling ratios, matching the four-subtype precision treatment framework (see **Table 1**). Meanwhile, NK cell-specific LNP delivery systems are expected to be further optimized to refine *in vivo in situ* cell engineering, reducing manufacturing complexity and improving treatment accessibility (119).

#### 6.3.5 Globally unified regulatory and quality control standards

Accumulation of multi-center clinical data is expected to promote the development of dedicated regulatory guidelines for AI-empowered CAR cell therapies, covering algorithm transparency, long-term safety surveillance and *in situ* engineering classification. Meanwhile, manufacturing cost optimization will comprehensively improve the accessibility of clinical treatment (120).

### 6.4 Evidence level stratification of core intervention strategies

Building on the future R&D roadmap outlined above, this section systematically stratifies all core technical strategies by evidence strength, applying the three-tier grading framework established in Section 2.4 to avoid overinterpretation of preclinical or conceptual findings. The full stratification is summarized in **Table 2**.

**Table 2:** Evidence level stratification of core interventions for dual-module CAR-NK therapy.

Broad category	Strategy category	Specific intervention	Core mechanism	Evidence level	Key limitations
Endogenous cellular engineering	Core dual-module CAR systems	KIR-based aCAR-iCAR system	AND-NOT logic gating; blocks trogocytic fratricide & off-target toxicity	Level 1	Restricted by HLA-I heterogeneity; increased manufacturing complexity
		NKG2A-based aCAR-iCAR system	HLA-E recognition; tunable ITIM inhibitory signaling	Level 2–3	Requires CD94 heterodimerization; risk of tonic inhibitory signaling
	AI-empowered design tools	Multi-omics target screening (GNN/Transformer)	Pan-cancer antigen prediction; immune escape risk evaluation	Level 2	Lacks dual-module CAR-NK-specific training datasets
		CAR structure & signaling optimization (CAR-Toner, AlphaFold)	Tonic signaling prediction; scFv affinity & stability tuning	Level 2	Most models not fine-tuned for NK cell-specific signaling networks
		Clinical digital twin & PK/PD modeling	Efficacy/toxicity prediction; individualized dosing optimization	Level 2	Limited real-world CAR-NK clinical data for model training
	Synergistic cell engineering	Multispecific CAR fusion	Reduced antigen escape; expanded target coverage	Level 2	Potential steric hindrance among multiple scFvs; increased signal balancing complexity
		Metabolic reprogramming & CISH/TGFBR2 editing	Enhanced TME stress resistance & *in vivo* persistence; amplified IL-15 responsiveness	Level 2	Long-term metabolic and genomic safety unclear; potential perturbation of endogenous NK signaling
Exogenous delivery & TME remodeling	Nanodelivery & TME remodeling tools	NK-specific mRNA-LNP delivery	Non-viral CAR transduction; *in vivo in situ* engineering	Level 2	Suboptimal *in vivo* targeting specificity; transient CAR expression; dual-module co-transfection efficiency unvalidated
		MMP-responsive ECM-degrading nanocarriers	Stromal barrier degradation; enhanced tumor infiltration	Level 2	Potential off-target matrix degradation risk
		Nanoparticle backpack IL-15 delivery	CAR-activatable cytokine release; reduced systemic toxicity	Level 2	Solid tumor TME release efficiency remains to be verified
Clinical translation framework	Clinical stratification framework	Four-subtype precision treatment strategy	Molecular stratification matched to cascade resistance mechanisms	Level 3	Lacks prospective clinical validation; no standardized biomarker cut-offs; inter-tumoral heterogeneity may complicate classification

Evidence levels are defined as follows, consistent with the grading framework established in Section 2.4.

Level 1: Directly and systematically validated in dual-module CAR-NK systems via *in vitro* and *in vivo* experiments.

Level 2: Robustly validated in adjacent biomedical fields or conventional CAR-T/CAR-NK platforms, with reasonable mechanistic extrapolation to dual-module systems.

Level 2–3: Denotes strategies where core mechanistic components are validated in adjacent fields, while functional validation specifically in dual-module CAR-NK systems remains pending.

Level 3: Conceptual or theoretical framework based on mechanistic analysis, with no direct experimental validation in CAR-NK settings.

All classifications reflect the current literature as of the search cutoff (December 31, 2025). For Level 2 strategies, translational readiness for dual-module CAR-NK therapy should be interpreted in conjunction with the specific limitations listed.

## 7 Summary and outlook

### 7.1 Summary

With the inherent advantages of low toxicity, MHC-unrestricted recognition, and allogeneic universal applicability, CAR-NK cells have emerged as a leading platform for off-the-shelf tumor immunotherapy. This therapy has well-documented efficacy in hematologic malignancies, yet its clinical translation in solid tumors remains hampered by interconnected drug resistance mechanisms.

This review systematically summarizes and analyzes the pathological process underlying CAR-NK therapy failure in solid tumors, and identifies three core bottlenecks: TME-mediated functional exhaustion, non-adaptive CAR structural defects, and trogocytosis-mediated immune escape. Building on these independent findings, we propose an integrative cascade resistance framework and further characterize the negative regulatory roles of the ICAM-1-LFA-1 and NKG2A-HLA-E axis in this process, aiming to provide a systematic perspective for understanding solid tumor drug resistance mechanisms in the field.

In response to the aforementioned pathological features of cascading drug resistance, this review elaborates on the aCAR-iCAR dual-module system, which is engineered on the basis of NK cells’ innate “missing self” signaling balance mechanism. This technology enables a conceptual paradigm shift in CAR design, from a “single activation switch” to a “precise signaling balancer”, and holds the potential to simultaneously address the three core drug resistance bottlenecks. The NKG2A-iCAR element can potentially block trogocytosis and off-target toxicity, and when combined with the three-tier safety switch system, establishes a comprehensive toxicity control framework.

In terms of technological empowerment, AI-driven full-chain rational design overcomes the R&D limitations of traditional empirical trial-and-error approaches; AI-nanosymbiont technology synergistically addresses the exogenous delivery and infiltration barriers of solid tumors; and the four-subtype precision treatment strategy enables individualized matching between tumor molecular profiles and clinical regimens. Together, these advances provide systematic, practical guidance for clinical translation. Single-target intervention alone is insufficient to reverse the cascading drug resistance in solid tumors, and multi-dimensional synergy between endogenous cell engineering and exogenous delivery technologies holds greater promise for overcoming these therapeutic bottlenecks.

### 7.2 Limitations of the proposed framework

Notably, the cascade resistance framework proposed in this review still has certain limitations. First, the proposed strict sequential causal relationship between the three core bottlenecks has not been directly validated by longitudinal dynamic studies or multi-node gene perturbation experiments; current supporting evidence is largely based on cross-sectional correlations and indirect deductions, and the exact causal sequence and amplification efficiency across different solid tumor subtypes remain to be elucidated. Second, the universality of this framework across solid tumor types requires further verification; the dominant resistance mechanism may vary significantly between tumor subtypes with distinct TME features and antigen expression profiles.

To address these gaps, future studies employing longitudinal single-cell profiling and sequential multi-node genetic perturbations in relevant *in vivo* models will be critical to validate and refine the causal relationships proposed in this framework.

### 7.3 Future outlook

Looking ahead, the field will need to focus on three core scientific questions: first, how to achieve AI-mediated dynamic regulation of the net balance between NK cell activating and inhibitory signals to adapt to the complex, dynamic changes of the TME; second, to build multi-omics integrative models that accurately recapitulate the non-linear intercellular interactions within the TME; third, to fully preserve the innate immune functions of NK cells while maximizing the antitumor activity of CAR engineering.

Although AI-driven dual-module CAR-NK therapy still faces multiple challenges, including scalable manufacturing, algorithm interpretability, and refinement of the regulatory framework, the deepening integration of synthetic biology, artificial intelligence, and nanomedicine means that this technical system holds the potential to overcome the efficacy bottleneck of solid tumor immunotherapy. It may provide novel therapeutic options for patients with relapsed/refractory solid tumors, and propel the field of tumor immunotherapy into a new era of precision and intelligence.
